# Placenta autophagy is closely associated with preeclampsia

**DOI:** 10.18632/aging.204436

**Published:** 2022-12-19

**Authors:** Chaomei Li, Wei Liu, Qunxiu Lao, Haiying Lu, Yingting Zhao

**Affiliations:** 1 Department of Maternity Centre, Southern Medical University Affiliated Maternal and Child Health Hospital of Foshan, Foshan 528000, Guangdong, China

**Keywords:** preeclampsia (PE), autophagy, random forest, nomogram, immune

## Abstract

The pathogenesis of preeclampsia (PE) is complex and placental internal homeostasis is regulated by cellular autophagy. However, there are fewer studies related to the role of placental autophagy in the pathogenesis of PE. The GSE75010 and GSE10588 datasets were downloaded from the gene expression omnibus (GEO) database. In the GSE75010 (test cohort), 103 differentially expressed genes (DEGs) were screened using “Limma” package, and 281 PE characteristic genes were screened by weighted gene coexpression network analysis (WGCNA). Combined with the autophagy gene set, a total of 5 autophagy-related hub genes were obtained. Three biomarkers (HK2, PLOD2, and TREM1) were then further screened by random forest(RF) model and least absolute shrinkage and selection operator(LASSO) algorithm as diagnostic of PE. In the unsupervised consensus clustering analysis, HK2, PLOD2, and TREM1 may be synergistically involved in hypoxia-induced autophagy and hypoxia-inducible factor 1(HIF-1) signaling pathway to induce PE. In addition, we constructed and evaluated a nomogram model for PE diagnosis using these three key diagnostic biomarkers, and the results showed that the model had significantly excellent predictive power (AUC values of GSE75010 and GSE10588 datasets were 0.869 and 0.876, respectively). In terms of immune infiltration, a higher proportion of T cells CD8, and a lower proportion of Macrophages M2 were found in PE placentas compared to normal tissue, and high expression of HK2, PLOD2, and TREM1 were accompanied by low levels of Macrophages M2 infiltration. HK2, PLOD2, and TREM1 may be associated with the development of pre-eclampsia, and their mechanisms of action in preeclampsia need to be further investigated.

## INTRODUCTION

Preeclampsia(PE) is a multisystem disease diagnosed after 20 weeks of gestation without a history of hypertension, accounting for 4.6% of pregnancy-related complications [1]. It is one of the most common causes of fetal growth restriction, stillbirth and maternal pregnancy-related deaths [2]. Current research has found that preventive measures, such as oral low-dose aspirin, for high-risk groups of PE can reduce the incidence of PE and preterm birth [3]. However, the development of PE cannot be accurately predicted by ultrasound in combination with biophysical parameters, so it is essential to find appropriate biomarkers to help aid the clinician in a comprehensive judgement.

Available studies suggest that the onset of PE is associated with poor placental formation, inadequate blood perfusion, excessive inflammatory activation, and endothelial cell damage [4]. Studies have shown that placental trophoblast autophagy is associated with the development of PE [5, 6]. Autophagy is the most fundamental life phenomenon in eukaryotes and can be triggered by a wide range of stresses. Through the catabolic process of lysosomes, damaged proteins, senescent or damaged organelles and other structures are degraded to maintain the stability of the intracellular environment [7]. In physiologically hypoxic early pregnancy placentas, enhanced levels of autophagy are observed to support trophoblast invasion and vascular remodeling and to protect trophoblasts from cell death caused by hypoxia or nutrient deficiency [8]. However, when too much damaged tissue accumulates beyond what autophagy can tolerate, it causes excessive cellular autophagy leading to autophagic death. On the one hand, autophagic death of placental trophoblast cells is induced to inhibit their ability to infiltrate, and on the other hand, autophagic death of endothelial cells is induced to inhibit angiogenesis, thus triggering a series of pathophysiological processes in PE [9, 10]. Li Gao et al. found that the level of autophagy in the placental tissue of PE patients was highly increased compared with that of normal pregnant women [6]. It is also noted that in PE patients, as the placental microenvironment is altered, a series of abnormal responses such as oxidative stress occurs, inducing excessive autophagy of trophoblast or endothelial cells, which in turn promotes the development of PE.

This suggests that the internal homeostasis of the placenta is regulated to some extent by the autophagic mechanism, and that dysfunctional autophagy can lead to disruption of placental homeostasis and consequently to the development of pregnancy complications such as PE. However, there is still a lack of systematic exploration of the pathogenesis of autophagy in PE. In this study, based on the gene expression omnibus (GEO) database, the R language was used to systematically evaluate the potential mechanisms of autophagy-related genes in PE. The autophagy-related genes were further screened as diagnostic biomarkers for PE patients, and diagnostic models were developed based on the biomarkers. Finally, the relationship between the biomarkers and immune cell infiltration was explored.

## MATERIALS AND METHODS

### Extraction of PE data based on GEO database

A large amount of genome-wide RNA expression microarray data is available in the GEO database (https://www.ncbi.nlm.nih.gov/geo/). The data used in this study were downloaded from the GSE75010 dataset [11] and the GSE10588 dataset [12]. The GSE75010 was used as the test cohort (including 80 PE placentas and 77 non-PE placentas) and the GSE10588 as the validation cohort (including 17 PE placentas and 26 non-PE placentas), as shown in Table 1. Information on the clinical data for each sample of the GSE75010 dataset is detailed in Supplementary File 1. First, the platform annotation information was downloaded to match gene probes to gene names, and when multiple probes identified the same gene, the mean was calculated to determine its expression, and when a gene was expressed in all samples at 0, the gene was removed. Then, based on the R software (version 4.1.2) “limma” package, the data were normalized again by the “quantile normalization” algorithm in the “normalizeBetweenArrays” function.

**Table 1:** GEO data collection table.

**Datasets**	**Accession**	**Platform**	**Cohort**	**PE samples**	**Normal samples**
Microarray	GSE75010	GPL6244	Test cohort	80	77
	GSE10588	GPL2986	Validation cohort	17	26

The autophagy-associated gene set was obtained from the Human Autophagy Database (HADb: http://autophagy.lu/) and the Gene Set Enrichment Analysis (GSEA: https://www.gsea-msigdb.org/gsea/index.jsp) autophagy-associated gene set, a total of 531 autophagy-associated genes were collected (Supplementary File 2).

### Weighted gene coexpression network analysis (WGCNA)

The scale-free weighted gene co-expression network of the GSE75010 dataset was constructed using the “WGCNA” toolkit [13] in R to identify co-expressed genes and modules associated with PE. A soft threshold is set so that the network approximates a scale-free network for subsequent network construction. Hierarchical clustering trees were used to identify gene modules, and hierarchical clustering based on a topological overlap matrix (TOM)-based dissimilarity measure (1-TOM) was used to construct the relevant gene modules. Pearson correlation coefficients were calculated to determine the correlation of each module with the disease, to obtain the module with the highest correlation with the disease and to obtain the genes within the module.

### Differentially expressed gene (DEG) identification and functional enrichment analysis

Next, DEGs were screened for differentially expressed genes between PE and normal placental tissue samples using the LIMMA package [14] in R with a setting of |Log2FC| > 0.5 and adjusted P-value < 0.05. The PE-related differential autophagy genes were obtained by taking the intersection of the gene set within the module most related to the PE, DEGs and autophagy-related genes. These genes were subjected to Gene ontology (GO) functional enrichment analysis using the “clusterProfiler” and “org.Hs.eg.db” packages [15].

### Diagnostic gene screening and diagnostic model construction

A random forest (RF) model and a support vector machine (SVM) model were established based on the PE- related differential autophagy genes in the GSE75010 dataset. The “DALEX” package in R language was used to analyze and compare the above two models, draw the residual distribution and generate the receiver operating characteristic (ROC) curve, so as to obtain the best model. Finally, the “randomForest” package in R language and the least absolute shrinkage and selection operator (LASSO) logistic regression [16] were used to screen out the corresponding diagnostic genes and take the intersection to obtain the PE diagnostic biomarkers.

A nomogram model was built to predict the occurrence of PE by using the “rms” package [17], and the predictive ability of the nomogram model was evaluated using the concordance index (C-index), calibration curve, and decision curve analysis (DCA). In addition, the area under the curve (AUC) values for each the PE diagnostic biomarkers were calculated to understand the value of these genes in the diagnosis of the PE, and to validate the expression and diagnostic value of these genes using the GSE10588 dataset. Finally, based on the clinical characteristics of GSE75010 dataset samples, the correlation between the PE diagnostic biomarkers expression and clinical characteristics was further explored.

### Consensus cluster analysis

Based on the diagnostic biomarkers expression in the PE samples of the GSE75010 dataset, the “ConsensusClusterPlus” package was used to cluster and type the PE samples, and the maximum cumulative distribution function (CDF) index was selected as the best k value [18]. Principal component analysis (PCA) was performed on the clustering results to screen out DEGs between clusters, and perform functional enrichment analysis to explore the potential regulatory mechanisms of PE diagnostic biomarkers in PE.

### Evaluation of immune cell infiltration

By using the CIBERSORT algorithm [19], the relative proportions of 22 infiltrating immune cells in each sample of the GSE75010 dataset were estimated and visualised by R software, and the abundance of immune cells was visualised and compared between the PE and Normal groups using the “vioplot” package. Finally, the correlation of each PE diagnostic biomarkers with the abundance of 22 infiltrating immune cells was determined by calculating the spearman correlation coefficient.

### Statistical analysis

All statistical analyses were carried out using R software (version 4.1.2). Differences in expression of selected biomarkers and immune cell infiltration between the normal and PE groups were compared using the Wilcoxon test. Spearman analysis of correlation between expression of selected biomarkers and immune cell infiltration. The diagnostic accuracy of the selected biomarkers and nomogram model was assessed by differentiating the AUC generated by the ROC. For all statistical methods, P-value<0.05 or adjust P-value<0.05 were considered a significant difference. In this paper, adjust P-value<0.05 was used for both differentially expressed genes (DEGs) and functional enrichment analysis, and P-value<0.05 was used for the rest of the analysis. Also, the false positive discovery rate <5% was used as the threshold for statistical significance. When performing multiple hypothesis tests, in order to avoid increasing the probability of making Type I errors, we use the Benjamini-Hochberg (BH) method to correct the P value to make the P value larger to control the number of false positives. The method is to sort all original P values from large to small, assign the largest P value as n, and assign the smallest P value as 1. Corrected P-value = original P-value * (n/i).

## RESULTS

### Identification and enrichment analysis of autophagy genes among key modules and DEGs

**Figure 1:**
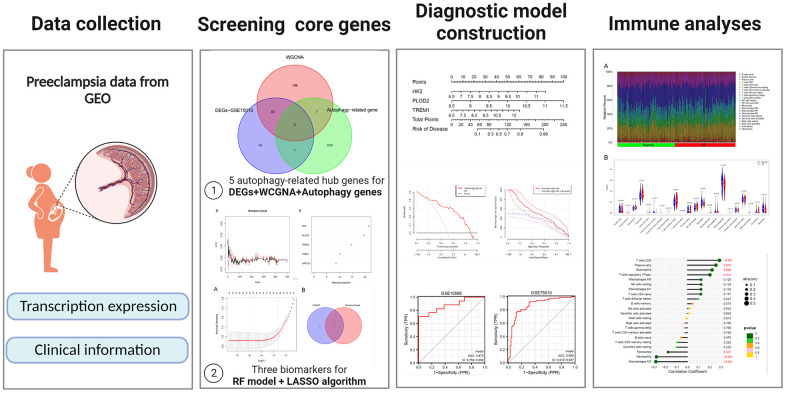
General overview of the study.

**Figure 2:**
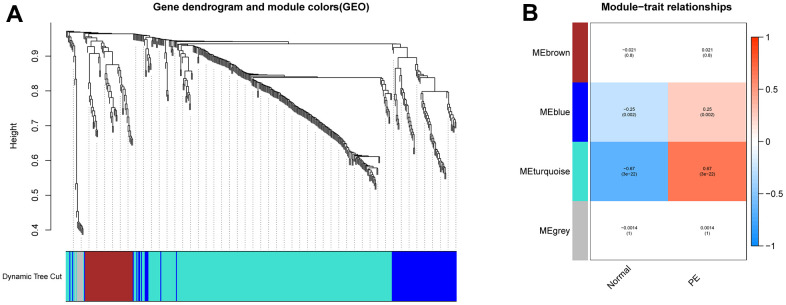
**Construction of WGCNA network.** (**A**) Screening the co -expression module of the PE. (**B**) Heatmap of the module-trait correlations. WGCNA, weighted gene coexpression network analysis. PE, preeclampsia.

**Figure 3:**
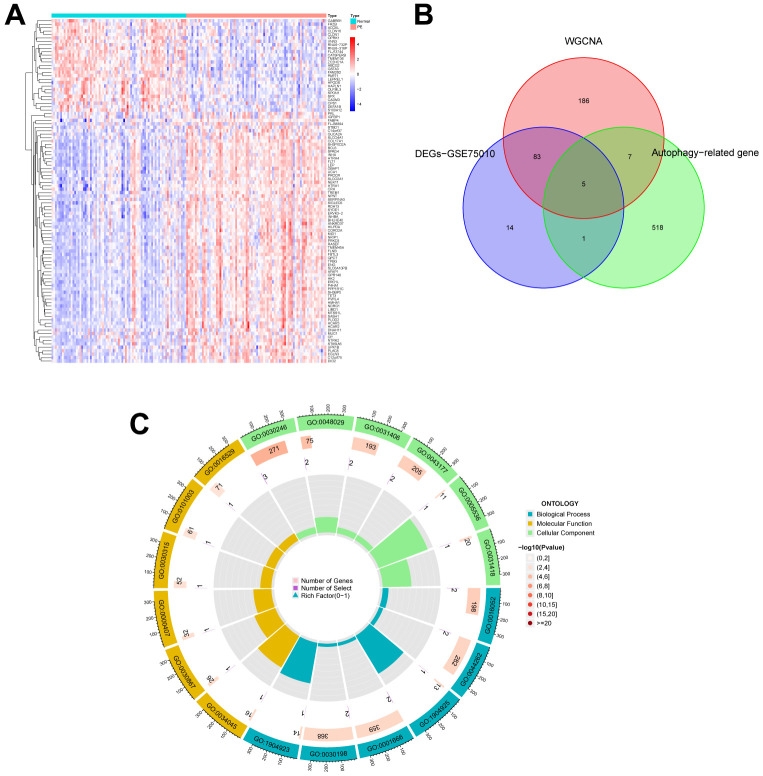
**Identification of autophagy genes among key modules and DEGs, and enrichment analysis.** (**A**) Heatmap plot of the DEGs in GSE75010. Blue represents down-regulation, red represents up-regulation, and the darker the color in the heatmap, the higher the significance. (**B**) Venn plot exhibiting the autophagy genes among key modules and DEGs, including HK2, PLOD2, TREM1, STBD1, and HAPLN1. (**C**) GO enrichment analysis of autophagy genes among key modules and DEGs. DEGs, differentially expressed genes. GO, gene ontology.

Figure 1 illustrates the workflow of the study. GSE75010 was downloaded from the GEO database, and the R software was used to construct the co-expression network. According to the scale-free fitting index of 0.9, the optimal soft threshold β=4 was determined (Supplementary Figure 1B). At this time, the average degree of connectivity of the network is relatively high and can contain enough information to construct a co-expression network (Figure 2A). Four gene modules were obtained, with the highest correlation of 0.67 between the turquoise module and PE (Figure 2B), and 281 genes within the module (Supplementary File 3). The GSE75010 dataset yielded 103 DEGs, of which 76 were up-regulated and 27 were down-regulated (Figure 3A and Supplementary File 4). Genes within the turquoise module, differentially expressed genes and autophagy-related genes were intersected to obtain the key 5 PE-related differential autophagy genes, including HK2, PLOD2, TREM1, STBD1, and HAPLN1 (Figure 3B). GO functional enrichment analysis mainly focused on regulation of autophagy of mitochondrion in response to mitochondrial depolarization, response to hypoxia, carbohydrate metabolic process, phagocytosis (Figure 2C and Supplementary Table 1). These results suggest that these genes may play an important role in PE through cellular activities such as autophagy, hypoxia, energy metabolism, and inflammation.

### Autophagy-related diagnostic biomarker identification and verification

**Figure 4:**
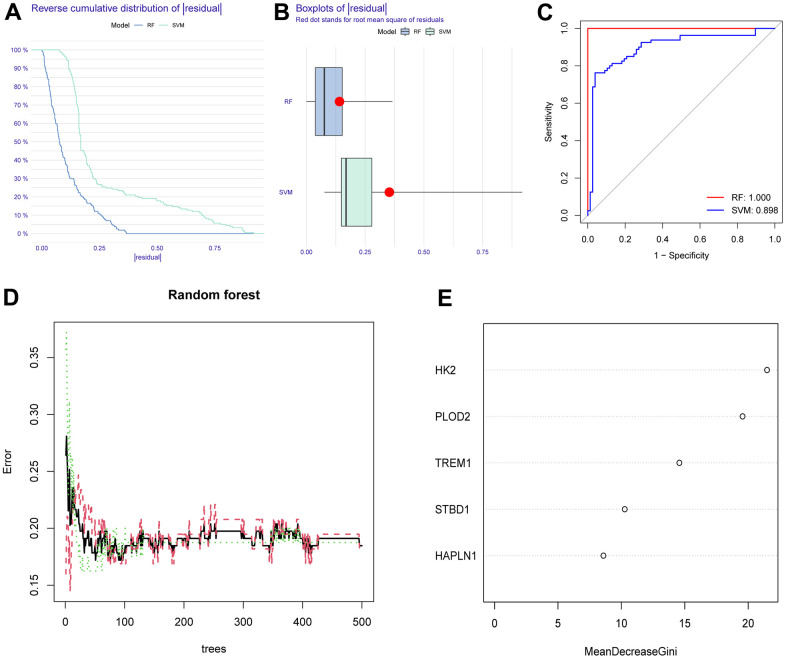
**Construction and assessment of RF and SVM model.** (**A**) Reverse cumulative residual distribution of RF and SVM model. (**B**) Boxplots of the residuals of RF and SVM model. (**C**) ROC of RF and SVM model. (**D**, **E**) RF algorithm of the sample. RF, random forest. SVM, support vector machine. ROC, receiver operating characteristic curve.

**Figure 5:**
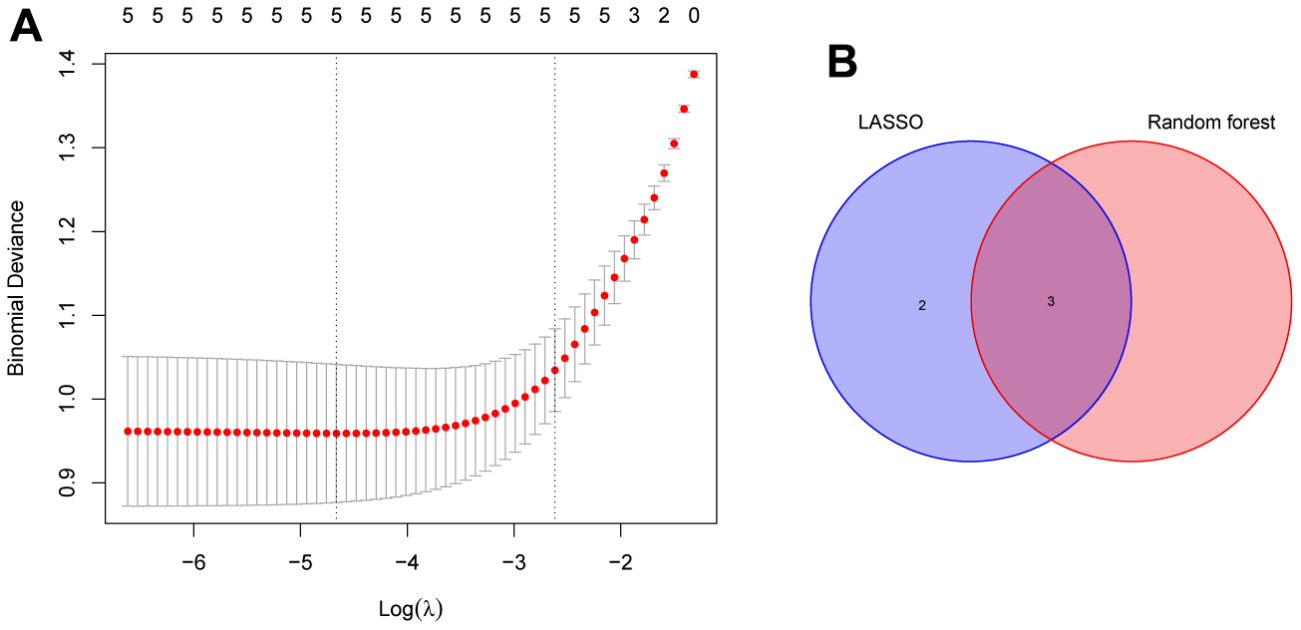
**Identification of the reliable autophagy biomarkers of PE.** (**A**) LASSO regression analysis of five autophagy genes among key modules and DEGs. (**B**) Venn plot exhibiting the reliable autophagy biomarkers among LASSO and RF model, including HK2, PLOD2, and TREM1. PE, preeclampsia. LASSO, least absolute shrinkage and selection operator. DEGs, differentially expressed genes. RF, random forest.

**Figure 6:**
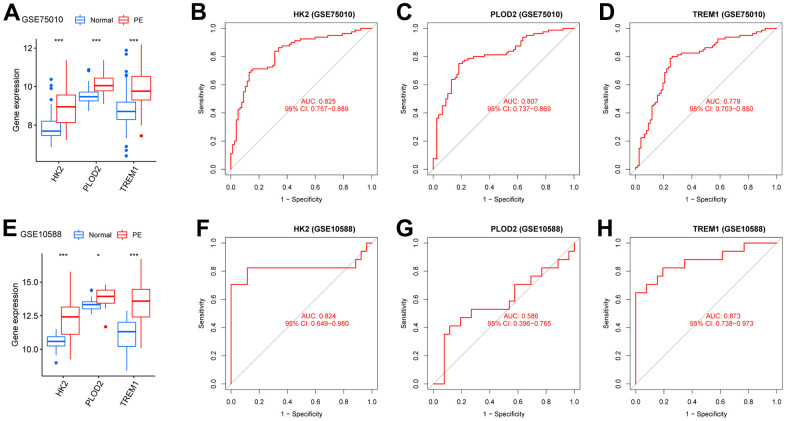
**Verification of the PE-related diagnostic biomarkers.** (**A**) The gene expression levels of HK2, PLOD2, and TREM1 in the test cohort (GSE75010). (**B**–**D**) ROC curves for evaluating the diagnostic ability of HK2, PLOD2, and TREM1 in the test cohort (GSE75010). (**E**) The gene expression levels of HK2, PLOD2, and TREM1 in the validation cohort (GSE10588). (**F**–**H**) ROC curves for evaluating the diagnostic ability of HK2, PLOD2, and TREM1 in the validation cohort (GSE10588). ROC, receiver operating characteristic curve.

To screen autophagy biomarkers with more diagnostic value, RF and SVM models were separately established. RF model has the lowest residual distribution compared with the SVM model (Figure 4A, 4B). ROC curve analysis indicated that the AUC value of the RF model was higher than that of the SVM model (Figure 4C). Based on these results, we believed that the RF model was the most suitable model. Based on the “randomForest” package, the 3 genes (HK2, PLOD2, and TREM1) with the highest importance scores in the RF model were selected for further analysis (Figure 4D, 4E). Next, five genes were extracted as candidate biomarkers by the LASSO regression algorithm (Figure 5A). The genes screened by the above two algorithms were then intersected by Venn diagram to obtain three reliable diagnostic biomarkers, including HK2, PLOD2, and TREM1 (Figure 5B). Compared with the normal group, the expression of HK2, PLOD2, and TREM1 was observed to be significantly upregulated in PE samples of the test cohort (GSE75010) (P < 0.05, Figure 6A), and the results were validated in the validation cohort (GSE10588) (P < 0.05, Figure 6E). To estimate disease prediction efficacy, ROC curves were performed and found that the AUC values for HK2, PLOD2, and TREM1 were 0.825, 0.807, and 0.779 (Figure 6B–6D) in the test cohort (GSE75010), and 0.824, 0.586, and 0.873 (Figure 6F, 6G) in the validation cohort (GSE10588), respectively. Interestingly, HK2, PLOD2, and TREM1 were significantly upregulated in the early-onset preeclampsia cohort (Supplementary Figure 2A), and TREM1 was also significantly upregulated in PE patients with concomitant hemolysis, elevated liver enzymes and low platelets (HELLP) complications (Supplementary Figure 2B).

### Establishment and assessment of a nomogram model for PE diagnosis

**Figure 7:**
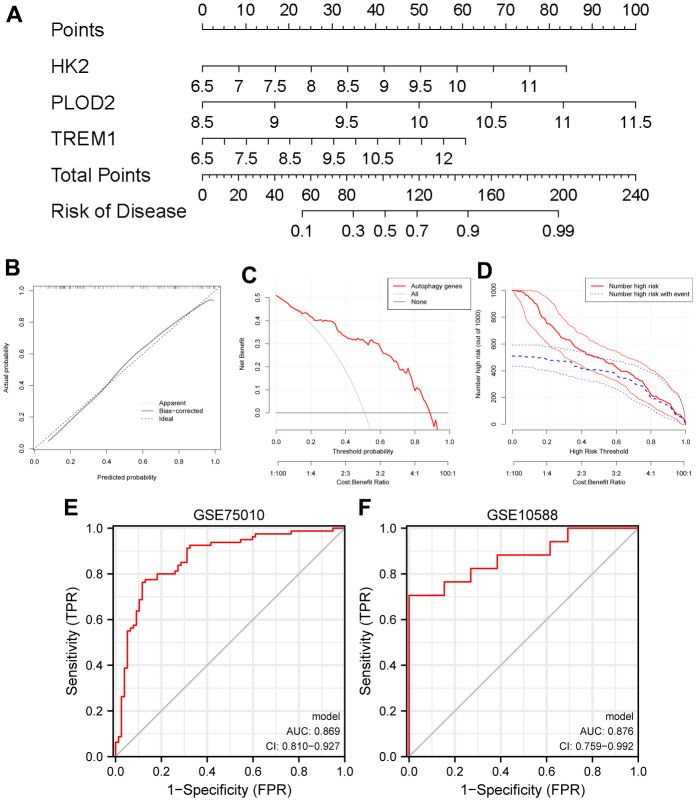
**Establishment of a nomogram model for PE diagnosis based on the test cohort (GSE75010).** (**A**) Nomogram to predict the occurrence of PE. (**B**) Calibration curve for the predictive power of the nomogram model. (**C**) DCA for the nomogram model. (**D**) Clinical impact curve to assess the nomogram model. (**E**, **F**) ROC curve to assess the model’s ability to diagnose PE in the test cohort (GSE75010) and the validation cohort (GSE10588). PE, preeclampsia. DCA, decision curve analysis. ROC, receiver operating characteristic curve.

Based on the expression of HK2, PLOD2, and TREM1 from the test cohort (GSE75010), the PE diagnostic nomogram model was established using the Rms package (Figure 7A). The error between the predicted and true event probabilities in the calibration curve had very small (Figure 7B), and the DCA indicated that the nomogram model had a higher clinical benefit than all (Figure 7C). At high risk thresholds from 0.2 to 1, the “Number high risk” curve and the “Number high risk with event” curve gradually tended to overlap (Figure 7D). In addition, this nomogram model showed high AUC values (0.869, 0.876; Figure 7E, 7F) in both the test cohort (GSE75010) and the validation cohort (GSE10588). These results indicate that this nomogram model has excellent predictive performance.

### Construction of unsupervised consensus clustering

**Figure 8:**
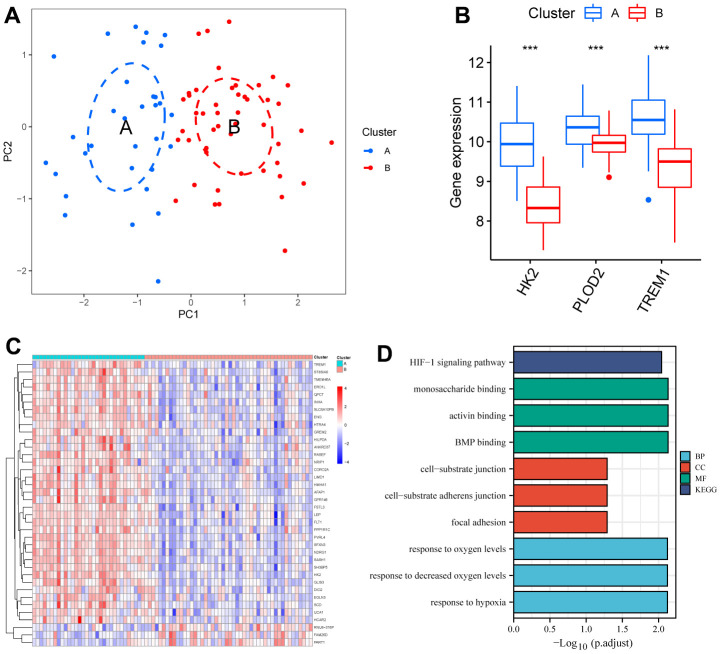
**Identification of two clusters using unsupervised consensus clustering.** (**A**) Principal component analysis of two clustered distributions. (**B**) Differences in gene expression levels of HK2, PLOD2, and TREM1 between the two clusters. (**C**) Heatmap plot of the DEGs between the two clusters. (**D**) GO and KEGG enrichment analysis of the DEGs. DEGs, differentially expressed genes. GO, gene ontology. KEGG, Kyoto Encyclopedia of Genes and Genomes.

The PE samples were clustered and typed using the “ConsensusClusterPlus” package based on the expression of HK2, PLOD2, and TREM1 from the test cohort (GSE75010). When k = 2, it has cluster stability (Supplementary Figure 3A, 3B), PE samples are divided into ClusterA (n = 32) and ClusterB (n = 48) (Supplementary Figure 3C), PCA also shows two subtype classifications better (Figure 8A). HK2, PLOD2, and TREM1 were significantly upregulated in ClusterA (P<0.05, Figure 8B). Screening between the two subtypes yielded 38 DEGs (Figure 8C). GO functional enrichment analysis focused on response to hypoxia, response to decreased oxygen levels, response to oxygen levels, etc. KEGG enrichment analysis showed that 38 DEGs were mainly involved in the HIF-1 signaling pathway (Figure 8D and Supplementary Table 2), and these results suggest that HK2, PLOD2, and TREM1 may play a key role in the PE process through hypoxia-induced autophagy.

### Analysis of immune cell infiltration

**Figure 9:**
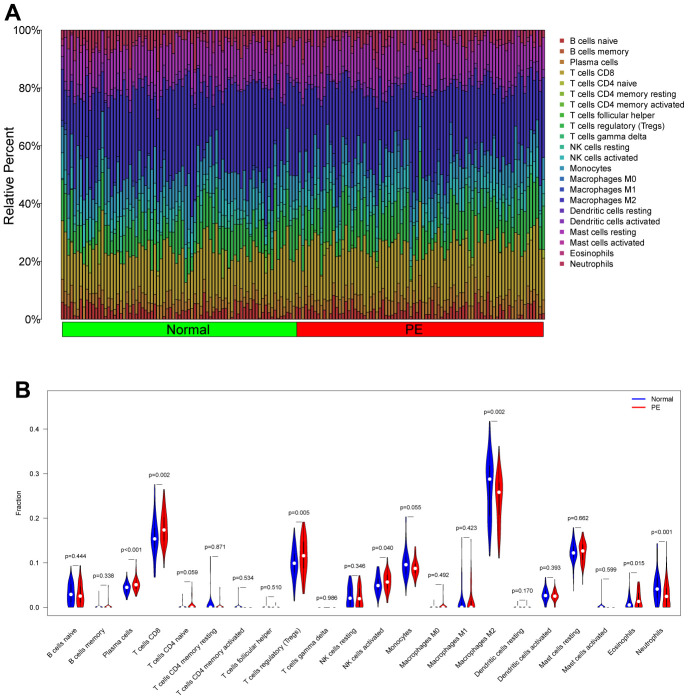
**Analysis of immune cell infiltration in the test cohort (GSE75010).** (**A**) Distribution map of infiltrated immune cells. (**B**) Differences of the infiltrated immune cells between the PE group and Normal group. PE, preeclampsia.

**Figure 10:**
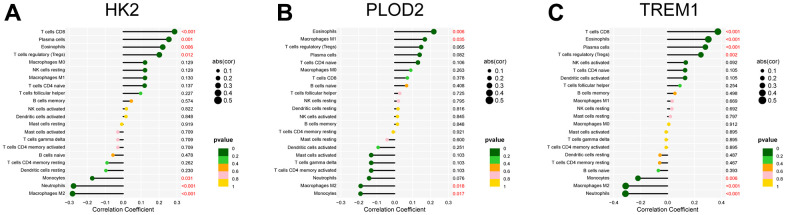
Correlation analysis between the infiltrated immune cells and the expression of the PE-related diagnostic biomarkers, including HK2 (**A**), PLOD2 (**B**), and TREM1 (**C**). PE, preeclampsia.

The “CIBERSORT” algorithm was used to estimate the abundance of immune cell infiltration in each sample of the GSE75010 dataset (Figure 9A). Compared to normal tissue samples, Plasma cells, T cells CD8, T cells regulatory (Tregs), NK cells activated and Eosinophils were more abundantly infiltrated in PE samples, while Macrophages M2 and Neutrophils were less abundantly infiltrated (P < 0.05, Figure 9B). Correlation analysis showed that HK2 was positively correlated with T cells CD8 (Cor= 0.286, P<0.001), Plasma cells (Cor=0.255, P=0.001), Eosinophils (Cor=0.220, P=0.006), T cells regulatory (Tregs) (Cor =0.201, P=0.012), and negatively correlated with Monocytes (Cor=-0.172, P=0.031), Neutrophils (Cor=-0.277, P<0.001), Macrophages M2 (Cor=-0.282 P<0.001); PLOD2 was positively correlated with Eosinophils (Cor= 0.218, P= 0.006), Macrophages M1 (Cor= 0.168, P= 0.035), and negatively correlated with Macrophages M2 (Cor= -0.189, P= 0.018), Monocytes (Cor= -0.190, P= 0.017); TREM1 was positively correlated with T cells CD8 (Cor= 0.374, P<0.001), Eosinophils (Cor= 0.303, P<0.001), Plasma cells (Cor= 0.281, P<0.001), T cells regulatory (Tregs) (Cor= 0.247, P=0.002), and negatively correlated with Monocytes (Cor= -0.220, P=0.006), Macrophages M2 (Cor= -0.311, P<0.001), Neutrophils (Cor= -0.313, P<0.001) (Figure 10).

## DISCUSSION

PE is one of the unique conditions of pregnancy that poses a serious threat to the life and health of the mother and baby. The only treatment is interruption of the pregnancy, but this may increase the risk of preterm complications for both mother and baby. Although screening indicators such as the biomarkers soluble fms-like tyrosine kinase 1 (sFlt-1) and placental growth factor (PlGF) have been used to predict PE by national and international scholars [20, 21], the predictive effect of these tests alone is not satisfactory, and there is an urgent need to find biomarkers with high specificity and sensitivity.

Studies have shown that autophagy, as an important mechanism for maintaining homeostasis within the placenta, is involved in its energy regulation, stress protection, immune regulation and other processes, maintaining the dynamic homeostasis of tissues and ensuring cellular activity for normal physiological functions of the placenta [22]. Disruption of placental homeostasis due to autophagy dysfunction can cause the development and exacerbation of PE [9]. Studies over the last decade or so have shown that autophagy also plays a key role in mobilizing various cellular energy and nutrient stores, including carbohydrates (glycophagy i.e., the autophagic degradation of glycogen) [23]. Glycophagy plays a critical role in maintaining energy homeostasis in many tissues, including heart, liver and skeletal muscle [24], however, the importance in preeclampsia remains unclear. In this study, five WGCNA screened differentially expressed autophagy-related genes were obtained, and the results showed that these genes were associated with behaviors such as carbohydrate catabolic, hypoxia and mitochondrial autophagy. The RF model and LASSO were further screened for three key diagnostic biomarkers (HK2, PLOD2, and TREM1). Hexokinase 2 (HK2) is a key enzyme in the glycolytic pathway and current studies have found that abnormal elevations in HK2 are associated with the development and malignant proliferation of a variety of tumors [25, 26]. Hou S et al. reported that HK2 expression was elevated in endometriosis tissues and that inhibition of HK2 expression effectively attenuated the migration, invasion, and proliferation of endometrial stromal cells [27]. Lv H et al. also found HK2 to be significantly upregulated in PE tissue [28]. TREM1 has been reported to be upregulated in pre-eclamptic placentas and to enhance trophoblast migration and invasion through activation of the NF-κB pathway [29]. However, a role for PLOD2 in PE has not been reported. We then used these three key diagnostic biomarkers to construct and evaluate a nomogram model for PE diagnosis, and the nomogram models have excellent predictive power based on the evaluation indicators (Calibration curve, DCA, Clinical impact curve, ROC curve).

In our cluster typing analysis HK2, PLOD2, and TREM1 may be synergistically involved in hypoxia-induced autophagy and the hypoxia-inducible factor 1(HIF-1) signaling pathway, contributing to the development and progression of pre-eclampsia. In normal placental development, physiological hypoxia increases the expression of hypoxia-inducible factor 1-alpha (HIF-1α), which activates autophagy via the PIK3 pathway and becomes a source of energy for trophoblast invasion, thereby maintaining intracellular homeostasis. It has been shown that multi-organ ischemia induces the production of HIF-1α further causing an increase in sFlt-1 levels and ultimately impairing placental function [30]. In PE cases, severe or persistent placental hypoxia accelerates the overexpression of HIF-1α in extravillous trophoblast (EVT) cells, leading to increased levels of soluble endothelial factor (sENG), which inhibits EVT autophagy. Autophagy damage prevents HIF-1α-mediated cellular energy depletion from being compensated in a timely manner, affecting energy balance, further impairing EVT invasiveness and vascular remodeling, resulting in superficial placental deposition and triggering PE [8, 31]. This is corroborated by the significantly lower adenosine triphosphate (ATP) levels in placentas with severe PE compared to normal placentas [32].

The inflammatory response is a recognized cause of PE and any local imbalance in the immune response may lead to abnormalities in placental structure or angiogenesis, contributing to the development of PE [33]. During placental insemination, the increase in immune cells such as NK cells and macrophages at the maternal-fetal interface not only has a local immune function, but also promotes the recruitment of trophoblast cells, the recasting of spiral arteries and the production of angiogenic factors, which play an important role in placental formation [34–36]. In the decidua of patients with preeclampsia, the expression of CD14+, CD163+ and Macrophages was increased, and the number of Macrophages M2 with anti-inflammatory effect was decreased. The decrease in the number of Macrophages M2 is related to the increased production of sflt-1, so it is speculated that the decrease in the number of Macrophages M2 may be related to the pathogenesis of preeclampsia [37], which is consistent with our findings. Interestingly, we also found that HK2, PLOD2, and TREM1 were all significantly and negatively correlated with Macrophages M2 infiltration levels. In addition, PE patients often suffer from immune imbalance due to inadequate trophoblast invasion and placental hypoxia, resulting in an increase and sequential activation of pro-inflammatory immune cells (e.g., CD8+ T cells) [38, 39]. We also observed elevated levels of placental CD8+ T cell infiltration in pre-eclampsia. However, our finding of higher numbers of Tregs in PE placental tissue is inconsistent with previous reports [40]. Overall, multiple infiltrating immune cells are collectively involved in the development and progression of PE.

There are also some limitations to our study. Firstly, this study is based on published data and key diagnostic biomarkers still need to be experimentally validated and their biological function in PE explored. Secondly, placental tissue needs to be obtained invasively, which carries a high risk, and the nomogram model is likely to be limited in clinical use.

## CONCLUSIONS

We identified HK2, PLOD2, and TREM1 as biomarkers for PE prediction using the WGCNA, RF, and LASSO algorithms, and thus developed a nomogram model for PE diagnosis with significantly better predictive power. The study also confirmed the potential association of infiltrating immune cells with the development of PE. These findings therefore provide a new perspective on the management and treatment of PE.

## Supplementary Material

Supplementary Figures

Supplementary Tables

Supplementary File 1

Supplementary File 2

Supplementary File 3

Supplementary File 4
