# Supplementary material for: Placenta autophagy is closely associated with preeclampsia
Source: Aging (Albany NY). 2022 Dec 19;15(24):15657–75. doi: 10.18632/aging.204436 (PMC10781466; doi:10.18632/aging.204436)
Supplement: Supplementary Figures [file aging-15-204436-s001.pdf]

SUPPLEMENTARY FIGURES

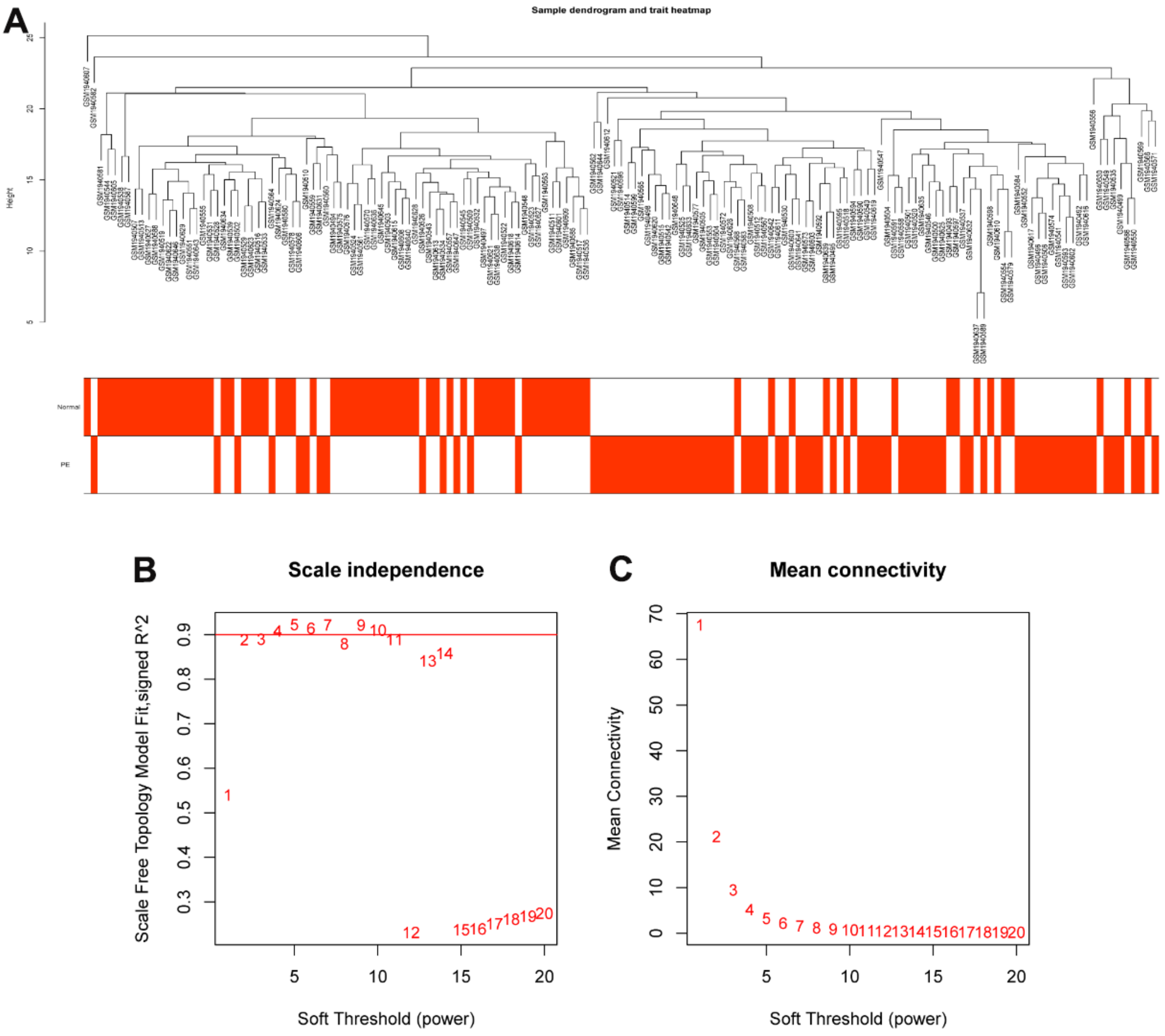

Supplementary Figure 1. Construction of WGCNA network. (**A**) Sample clustering of GSE75010. (**B**) Soft threshold determination. (**C**) Analysis of the mean connectivity for various soft-thresholding powers. WGCNA, weighted gene coexpression network analysis.

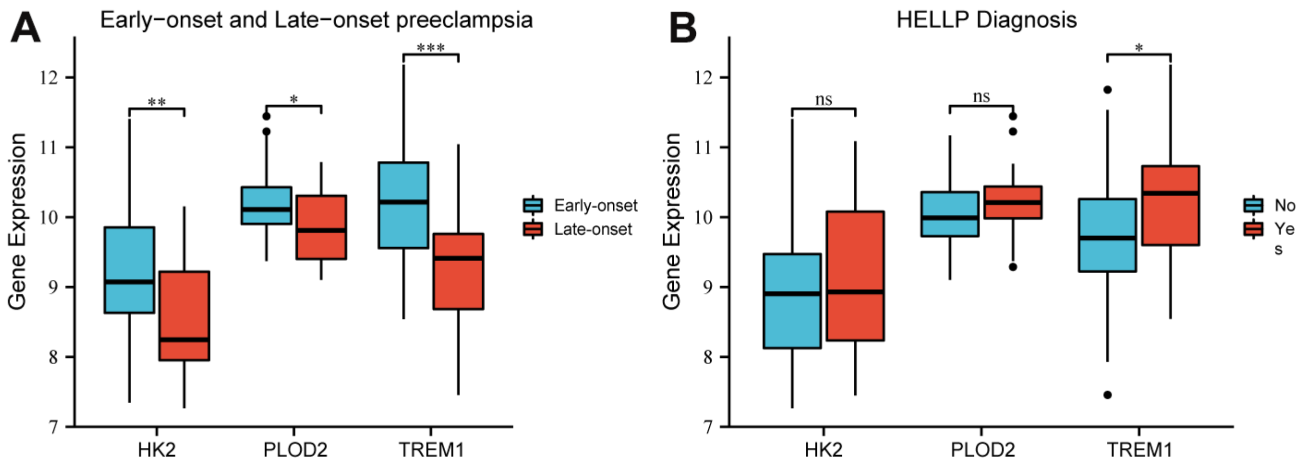

Supplementary Figure 2. Correlation analysis between the expression of the PE-related diagnostic biomarkers and clinical features, including preeclampsia staging (**A**) and HELLP syndrome (**B**). PE, preeclampsia. HELLP, hemolysis, elevated liver enzymes and low platelets.

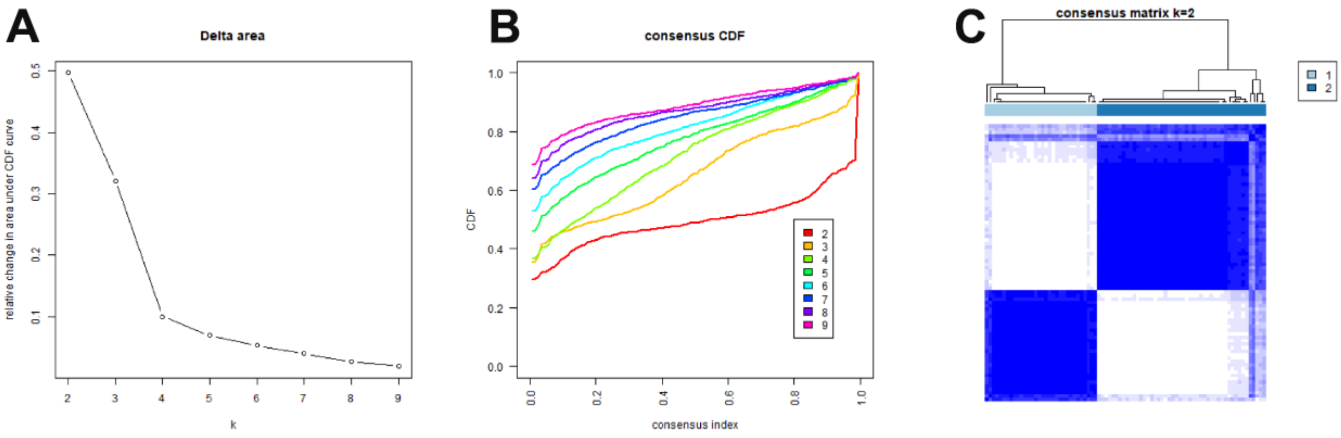

Supplementary Figure 3. Unsupervised consensus clustering in the test cohort (GSE75010). (**A**) Delta area curve of consensus clustering for k = 2 to 9. (**B**) CDF for k = 2 to 9. (**C**) Heatmap showing the two clusters of PE samples. CDF, cumulative distributive function. PE, preeclampsia.
