# Supplementary material for: Placenta autophagy is closely associated with preeclampsia
Source: Aging (Albany NY). 2022 Dec 19;15(24):15657–75. doi: 10.18632/aging.204436 (PMC10781466; doi:10.18632/aging.204436)
Supplement: Supplementary Tables [file aging-15-204436-s002.pdf]

SUPPLEMENTARY TABLES

Supplementary Table 1. GO functional enrichment analysis of the PE-related differential autophagy genes.

ONTOLOGY	ID	Description	pvalue	p.adjust	qvalue
BP	GO:0016052	carbohydrate catabolic process	0.001096	0.036522	0.011503
BP	GO:0044262	cellular carbohydrate metabolic process	0.002206	0.036522	0.011503
BP	GO:1904925	positive regulation of autophagy of mitochondrion in response to mitochondrial depolarization	0.003477	0.036522	0.011503
BP	GO:0001666	response to hypoxia	0.003548	0.036522	0.011503
BP	GO:0030198	extracellular matrix organization	0.003725	0.036522	0.011503
BP	GO:1904923	regulation of autophagy of mitochondrion in response to mitochondrial depolarization	0.003744	0.036522	0.011503
CC	GO:0034045	phagophore assembly site membrane	0.004051	0.037995	0.007499
CC	GO:0030867	rough endoplasmic reticulum membrane	0.006577	0.037995	0.007499
CC	GO:0000407	phagophore assembly site	0.008089	0.037995	0.007499
CC	GO:0030315	T-tubule	0.013119	0.037995	0.007499
CC	GO:0101003	ficolin-1-rich granule membrane	0.015375	0.037995	0.007499
CC	GO:0016529	sarcoplasmic reticulum	0.017877	0.037995	0.007499
MF	GO:0030246	carbohydrate binding	3.47E-05	0.00066	7.31E-05
MF	GO:0048029	monosaccharide binding	0.000176	0.00167	0.000185
MF	GO:0031406	carboxylic acid binding	0.001158	0.0062	0.000687
MF	GO:0043177	organic acid binding	0.001305	0.0062	0.000687
MF	GO:0005536	glucose binding	0.003105	0.011797	0.001307
MF	GO:0031418	L-ascorbic acid binding	0.005639	0.013092	0.001451

Supplementary Table 2. Functional enrichment analysis of the DEGs between the two clusters.

Ontology	ID	Description	pvalue	p.adjust	qvalue
BP	GO:0001666	response to hypoxia	1.58e-05	0.008	0.006
BP	GO:0036293	response to decreased oxygen levels	1.88e-05	0.008	0.006
BP	GO:0070482	response to oxygen levels	2.68e-05	0.008	0.006
CC	GO:0005925	focal adhesion	0.003	0.051	0.051
CC	GO:0005924	cell-substrate adherens junction	0.003	0.051	0.051
CC	GO:0030055	cell-substrate junction	0.003	0.051	0.051
MF	GO:0036122	BMP binding	1.32e-04	0.007	0.005
MF	GO:0048185	activin binding	2.17e-04	0.007	0.005
MF	GO:0048029	monosaccharide binding	2.22e-04	0.007	0.005
KEGG	hsa04066	HIF-1 signaling pathway	2.68e-04	0.009	0.007
